# Aberrant 5’-CpG Methylation of Cord Blood TNFα Associated with Maternal Exposure to Polybrominated Diphenyl Ethers

**DOI:** 10.1371/journal.pone.0138815

**Published:** 2015-09-25

**Authors:** Tyna Dao, Xiumei Hong, Xiaobin Wang, Wan-Yee Tang

**Affiliations:** 1 Department of Environmental Health Sciences, Johns Hopkins Bloomberg School of Public Health, Baltimore, Maryland, United States of America; 2 Department of Human Population and Reproductive Health, Johns Hopkins Bloomberg School of Public Health, Baltimore, Maryland, United States of America; Massachusetts General Hospital, UNITED STATES

## Abstract

Growing evidence suggests that maternal exposures to endocrine disrupting chemicals during pregnancy may lead to poor pregnancy outcomes and increased fetal susceptibility to adult diseases. Polybrominated diphenyl ethers (PBDEs), which are ubiquitously used flame-retardants, could leach into the environment; and become persistent organic pollutants via bioaccumulation. In the United States, blood PBDE levels in adults range from 30–100 ng/g- lipid but the alarming health concern revolves around children who have reported blood PBDE levels 3 to 9-fold higher than adults. PBDEs disrupt endocrine, immune, reproductive and nervous systems. However, the mechanism underlying its adverse health effect is not fully understood. Epigenetics is a possible biological mechanism underlying maternal exposure-child health outcomes by regulating gene expression without changes in the DNA sequence. We sought to examine the relationship between maternal exposure to environmental PBDEs and promoter methylation of a proinflammatory gene, *tumor necrosis factor alpha* (*TNFα*). We measured the maternal blood PBDE levels and cord blood *TNFα* promoter methylation levels on 46 paired samples of maternal and cord blood from the Boston Birth Cohort (BBC). We showed that decreased cord blood *TNFα* methylation associated with high maternal PBDE47 exposure. CpG site-specific methylation showed significantly hypomethylation in the girl whose mother has a high blood PBDE47 level. Consistently, decreased *TNFα* methylation associated with an increase in TNFα protein level in cord blood. In conclusion, our finding provided evidence that *in utero* exposure to PBDEs may epigenetically reprogram the offspring’s immunological response through promoter methylation of a proinflammatory gene.

## Introduction

Polybrominated diphenyl ethers (PBDEs), known as endocrine disrupting compounds, affecting the endocrine, immune, reproductive and nervous systems [1]. PBDEs, until recently, are ubiquitously used flame-retardants, in many consumer products, that have leached into the environment and bioaccumulated to become persistent organic pollutants (POPs) [2, 3]. There are over 209 congeners of PBDEs, but five are significant to humans, since they account for 90% of the total body burden; PBDE-47, -99, -100, -153 and -154 [2]. Human PBDE exposure occurs through multiple exposure pathways; inhalation, ingestion and dermal contact [4, 5]. Many of the lower PBDE congeners are lipophilic and accumulate in fatty tissues. They can easily cross the placenta into the fetal circulation [6]. Nursing mothers can transfer PBDEs to infants through breast milk [4]. PBDEs also accumulate in fetal lipid-rich tissues such as brain, liver and adipose. PBDEs are persistent in the body with estimated human half-lives of 2–12 years [6]. Epidemiological and experimental studies suggest that PBDEs are developmental neurotoxicants, at least partly impairing thyroid hormone homeostasis, oxidative damage or dysregulation of neurotransmitter signaling in the brain [7].

2,2’,4,4’-Tetrabromodiphenyl ether (PBDE47) accounts for 50% of the PBDE body burden [7] and is the PBDE congener found at the highest levels in human serum and breast milk [8–10]. Female mice exposed prenatally or postnatally to repeated low doses of PBDE47 resulted in the maternal transfer of PBDE47 from dams to the fetus blood, brain, liver and fat tissues. The offspring showed growth retardation and behavioral deficiencies [3, 5] [11]. These animal findings are supported by epidemiological evidence of the relationship between PBDE47 levels and cognitive delay [12, 13]. Furthermore, animals exposed to a commercial PBDE mixture (PBDE47 is the dominant congener) showed decreased lymphocyte proliferation and antibody production [14], suggesting that PBDEs may pose a risk of immunotoxicity on specific target tissues. PBDE47 is postulated to disrupt thyroid function because it shares a similar structure to thyroid hormone [1]. However, studies have reported that PBDE47 does not interact with the thyroid hormone receptor [15] suggesting that alternative mechanisms may underlie the toxicity of PBDE47 in the cells.

It has been proposed that maternal PBDE exposure induces epigenetic reprogramming of the offspring and associates with increased disease risks in children. The “Barker Hypothesis” postulated that organs undergo developmental programming in the womb that predetermines subsequent physiological and metabolic adaptations during childhood or as adults [16, 17]. Epigenetics now underpins the developmental reprogramming by demonstrating the molecular relationship between the exposure to environmental pollutants/toxicants and gene expression changes that influence disease susceptibility [18–23]. Epigenetics mechanisms (DNA methylation, histone modifications and microRNAs) act singularly or conjointly to regulate gene expression without altering DNA sequences in response to environmental exposures. Therefore, they produce an array of unique phenotypes that control cell differentiation and organ development. DNA methylation is the covalent addition of a methyl group from S-adenosyl-methionine (SAM), by DNA methyltransferases (DNMTs), to the fifth position of cytosine in CpG dinucleotides to generate 5-methylcytosine (5-mC). DNA methylation at CpG dinucleotides mostly occurs in the 5’ flanking region and often associates with repression of gene activity. The biological contribution of DNA methylation may vary according to the position of the specific CpG sites relative to the transcription unit and the binding of transcriptional factors at the promoter [24]. Decreased global DNA methylation associates with high blood POPs levels [25]. A longitudinal birth cohort study, Center for the Health Assessment of Mothers and Children of Salinas (CHAMACOS) reported increased parental exposure to PBDEs associates with decreased global DNA methylation, measured on the *Alu (Arthrobacter luteus)* and *LINE-1 (Long Interspersed Elements)* sequences, in the cord blood of newborns [26]. By stratifying the children by sex, they revealed that there is a dynamic relationship between the PBDEs exposure and global DNA methylation changes that result from differences in sex, age, exposure-dose and multiple exposures. Mice prenatally exposed to PBDE47 showed decreased global methylation in their brains that persists into adulthood [1]. Recently, low perinatal PBDE47 exposure has been demonstrated to influence the methylation of mitochondrial cytochrome c oxidase (*Mt-co2*) and genes related to brain function (*Bdnf* and *Nr3c1*) [27]. All in all, it suggests exposure to PBDE47 may affect cell and tissue functions by regulating gene expression through DNA methylation.

Exposure to PBDEs in rodents was shown to impair the immune function [28, 29]. Their interaction with the circulating immune cells may be driving the immunological signals to the target tissues. It could be expected that early life exposure to PBDEs may disrupt immune cell functions via epigenetic modifications of gene regulation. Once the epigenetic program is set, epigenetic disruption of the immune system can persist throughout life, resulting in increased susceptibility to inflammation-related diseases later in life. Additionally, dysregulation of immune responses has been suggested to be associated with neurological dysfunctions. Production of cytokines like Interleukin 1 beta (*IL1β*) and Tumor Necrosis Factor alpha (*TNFα*) from the peripheral blood cells are extensively studied and suggested to be affecting the central nervous system [30–32]. However, there are no studies investigating the relationship between the PBDEs exposure and DNA methylation patterns of these two cytokines. We first utilized an *in vitro* system to test the epigenetic effect of PBDEs on these cytokines by exposing peripheral blood mononuclear cells (PBMCs) to PBDE47. *TNFα* methylation, not *IL1β*, showed a linear relationship to the doses of PBDEs. As a proof of concept, we utilized paired maternal and cord blood samples from the Boston Birth Cohort (BBC) and examined if maternal PBDE47 exposure was associated with cord blood *TNFα* promoter methylation. Our findings may provide the insight to understand the adverse effects of maternal PBDEs exposure on human health through novel epigenetic mechanisms, affecting immunity and inflammation and resulting in higher susceptibility of immune diseases like food allergy, asthma, or other metabolic symptoms [33, 34].

## Materials and Methods

### Boston Birth Cohort (BBC)

This study included a subset of mother-infants pairs from the BBC, a cohort consisting of multiethnic mother-infant pairs (predominantly African American) enrolled 24 to 72 hours post delivery and followed prospectively from birth onward as detailed previously [34–36]. We collected the biospecimens including maternal blood and cord blood just after delivery. We obtained written informed consent from each mother. The study protocol was approved by the Institutional Review Boards (IRB) of Boston University Medical Center and by the IRB of Johns Hopkins University Bloomberg School of Public Health.

We measured the concentrations of lipid normalized to PBDEs in peripartum maternal serum samples of the BBC (Table 1). The median level of PBDE47 (12.1ng/g-lipid) was used as the rough cut-off for sample selection. To select samples for the high-level maternal PBDE47 exposed group, we sorted all the available samples and chose samples with PBDE47 levels higher than the median and with available clinical data and DNA samples. Similarly, to select samples for the low-level maternal PBDE47 exposed group, we sorted all the available samples and chose samples with the PBDE47 level lower than the median and with available clinical data and DNA samples. As a result, subjects in the low-level PBDE47 exposed group have maternal PBDE47 < 8.4 ng/g-lipid (about 36^th^ percentile in the BBC) and subjects in the high-level PBDE47 exposed group have maternal PBDE47 > 20 ng/g-lipid (about 70^th^ percentile in the BBC). The distribution of PBDE47 level in each group was illustrated in Table 2. The subjects in the low (n = 23) and high (n = 23)–level PBDE47 exposed groups also fall into the same low and high-level of the sum of all PBDEs measured.

**Table 1:** Concentrations of lipid normalized PBDEs in peripartum maternal serum samples of the BBC.

	Level (ng/g-lipid)			% Detection	
Congener	P25	P50	P75		Range
PBDE17	LOD	LOD	LOD	4.5	LOD-1.8
PBDE28	LOD	0.6	1.3	56.3	LOD-6.9
PBDE47	5.3	12.1	24.6	87.5	LOD-192.0
PBDE66	LOD	LOD	LOD	8.1	LOD-8.2
PBDE85	LOD	LOD	0.8	41.3	LOD-7.5
PBDE99	LOD	LOD	5.7	46.6	LOD-72.3
PBDE100	1.3	2.4	5	90.7	LOD-35.6
PBDE153	1.2	2.4	4.6	85	LOD-38.6
PBDE154	LOD	LOD	0.5	31.2	LOD-6.7
PBDE183	LOD	LOD	0.7	42.9	LOD-2.4
Total PBDEs	9.75	20.85	44.75	-	LOD-335.0

P, percentile; LOD, Lowest of Detection.

**Table 2:** Distribution of PBDE47 level in the low vs high maternal PBDE47 exposure group.

			Level (ng/g-lipid)				
Sex	PBDE47 exposure group	N	P25	P50	P75	Minimum	Maximum
**Girls**	Low	9	0	0	3.8	0	7.4
	High	12	38.6	57	104.7	20.8	175
**Boys**	Low	14	0	3.1	7.7	0	8.4
	High	11	31.7	35.4	44.9	20.6	192
**Total**	Low	23	1.2	0	5.7	0	8.4
	High	23	31.9	43.8	93.3	20.6	192

P, Percentile.

A total of 46 mother-infant pairs were selected for this study. In the low maternal PBDE47 group, PBDE47 level ranged from 0–8.4 ng/g-lipid (boys: 0–8.4 ng/g-lipid; girls: 0–7.4 ng/g-lipid). In the high maternal PBDE47 group, PBDE47 level ranged from 20.6–192.0 ng/g-lipid (boys: 20.6–192 ng/g-lipid, girls: 20.8–175.0 ng/g-lipid).

### PBDEs measurement in maternal serum

Maternal serum samples from each subject were prepared for shipment using Center for Disease Control (CDC)’s protocols and were sent to the CDC in Atlanta for PBDE measurement using an established and validated PBDE assay. The analytical method and quality control procedures have been described previously [37]. The method used for sample processing included automatic fortification of the samples with internal standards as well as addition of formic acid and water for denaturation and dilution of the samples using a Gilson 215 liquid handler (Gilson Inc.; Middleton, WI). The samples were thereafter extracted by solid phase extraction (SPE) using a Rapid Trace (Caliper Life Sciences; Hopkinton, MA) modular SPE system. Removal of co-extracted lipids was performed on a silica/sulfuric acid column using the Rapid Trace equipment for automation. Final analytical determination of the target analytes was performed by gas chromatography isotope dilution high resolution mass spectrometry (GC-IDHRMS) employing a MAT95XP (ThermoFinnigan MAT, Bremen, Germany) instrument. The samples were analyzed for 10 PBDE congeners: PBDE-17, -28, -47, -66, -85, -99, -100, -153, -154, -183 (comprising tri-, tetra-, penta-, hexa- and hepta-brominated congeners, but not deca).

### Measurement of TNFα in cord blood serum sample

Cord blood sample was collected at delivery, using a BD Vacutainer® Plus Plastic K20EDTA tube (purple top). The sample was centrifuged at 2500g (4°C) for 10 minutes. Cord blood serum (the supernatant) was split into 3 aliquots and stored at −80°C. Cord blood cell fraction was transferred into a single cryotube and frozen at -80°C if DNA isolation was not performed immediately after collection. Serum sample was analyzed simultaneously for TNFα by immunoassay using flowmetric Luminex xMAP technology (Luminex Corp, Austin, TX), as reported previously [38].

### DNA isolation of cord blood leukocytes

Genomic DNA was extracted from cord blood leukocytes and quantified with SpectraMax M2 (www.moleculardevices.com). In brief, the cord blood cell fraction was subject to red blood cell (RBC) lysis and shaken for 10 minutes before centrifugation at 3000g (4°C) for 10 minutes. The cell pellet presented as the leukocytes and subjected to DNA isolation to collect the genomic DNA.

### Bisulfite genomic sequencing

**Fig 1:**
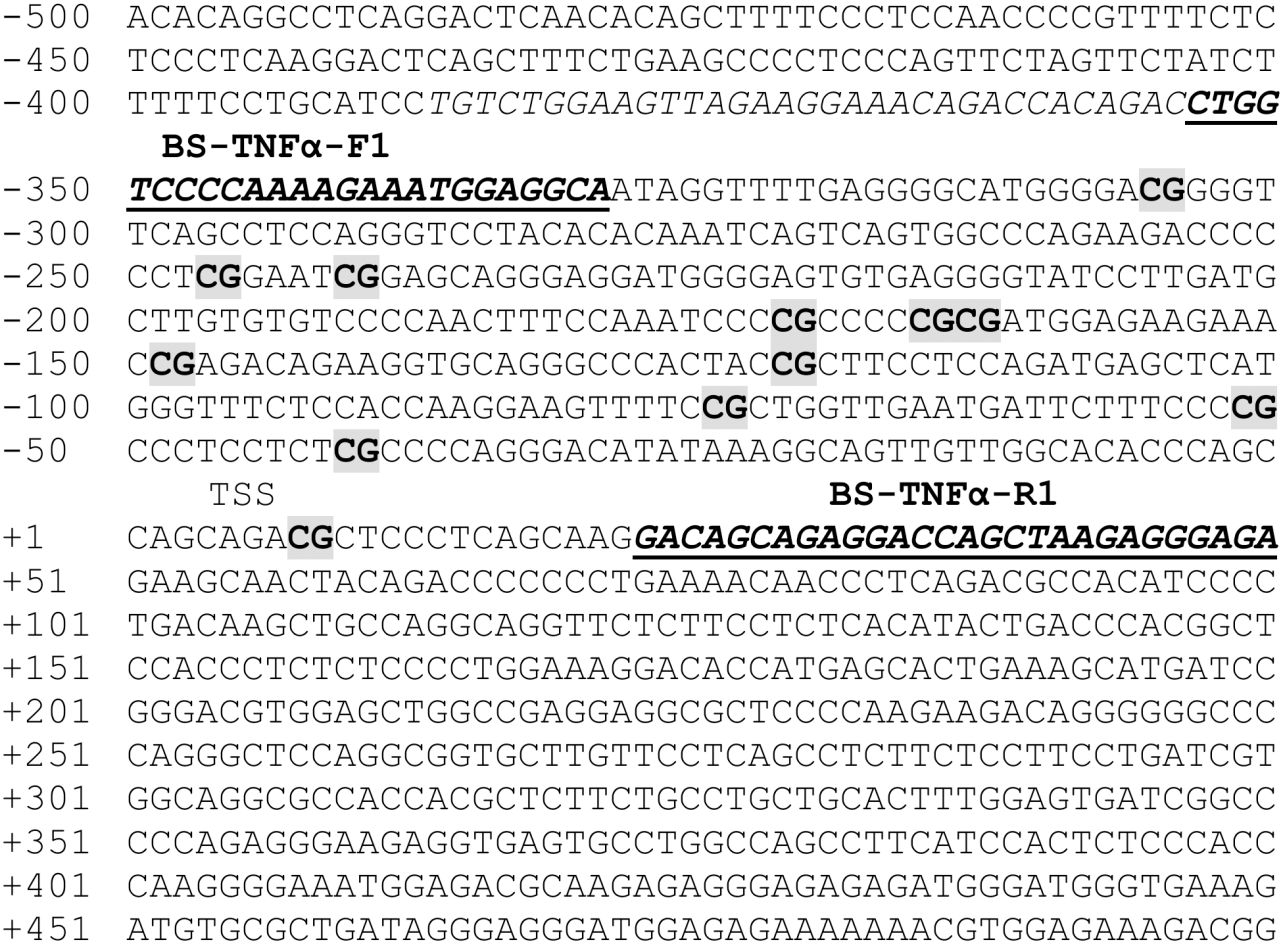
Genomic DNA sequence of the 5’ flanking region of TNFα promoter. The sequence of the primers used for bisulfite genomic sequencing (BS- TNFα-F1, BS- TNFα-R1) are underlined and in italic bold type. There are a total of 12 CpG nucleotides (shaded in gray) located at the promoter region of TNFα encompassing the transcription start site (TSS).

Genomic DNA (200ng) extracted from cord blood leukocytes was bisulfite-treated (EZ DNA methylation kit, Zymo Research) before PCR. The CpG rich region of the 5’ promoter region of *TNFα* (NC_000006.11: 31541344–31545344) was revealed by analyses of genomic sequences at MethPrimer (http://www.urogene.org/methprimer/) (Fig 1). Primers were designed to amplify a 404 base pair fragment (-354 to +50) including a total of 12 CpG sites from bisulfite-treated DNA: hBS-TNFα-F1:5’-TTGGTTTTTAAAAGAAATGGAGGTA-3’; hBS-TNFα-R1: 5’-TCTCCCTCTTAACTAATCCTCTACTATC-3’. PCR, using GoTaq PCR mix (Promega, MO), was performed on the bisulfite-treated DNA (95°C for 10 minutes, 35 cycles of 95°C for 30 seconds, 60°C for 1 minute and 72°C for 2 minutes, followed by an extension at 72°C for 12 minutes). PCR products were purified (GeneJET Gel Extraction Kit, Thermo Scientific, NY) and subcloned into pCR 2.1 vector (Invitrogen, CA). 4–6 clones from each sample were sequenced (Macrogen, MD) to obtain direct measures of DNA methylation at each CpG site in the *TNFα* promoter region. Sequencing data was analyzed with the BiQ analyser. Genomic DNA with 0 and 100% methylation was used as a quality control to ensure bisulfite conversion quality on sample.

### Data analysis

**Fig 2:**
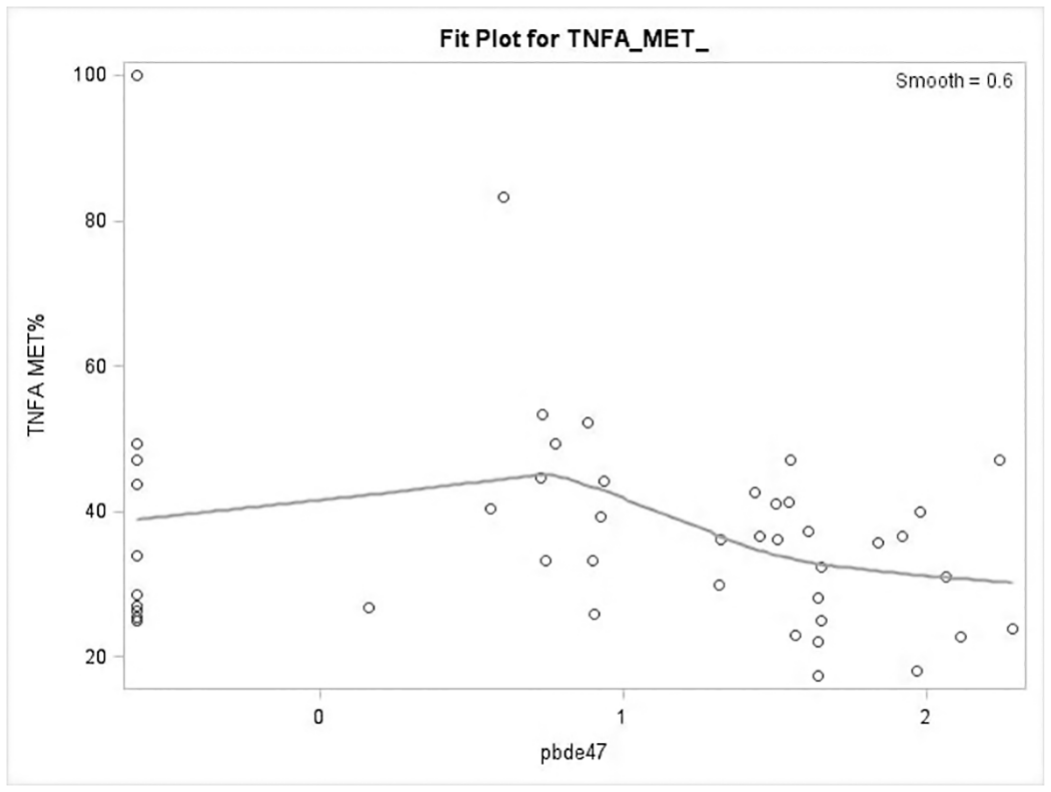
A scatter plot showing the relationship between the average % methylation of the *TNFα* promoter and log10-transformed maternal blood PBDE47 level. Methylation of each CpG site of the *TNFɑ* promoter was assayed in 46 cord blood DNA samples by bisulfite genomic sequencing. Average % methylation of the *TNFɑ* promoter (y-axis) was calculated by taking an average of the methylation level of a total 12 CpG sites within the *TNFɑ* promoter region.

**Fig 3:**
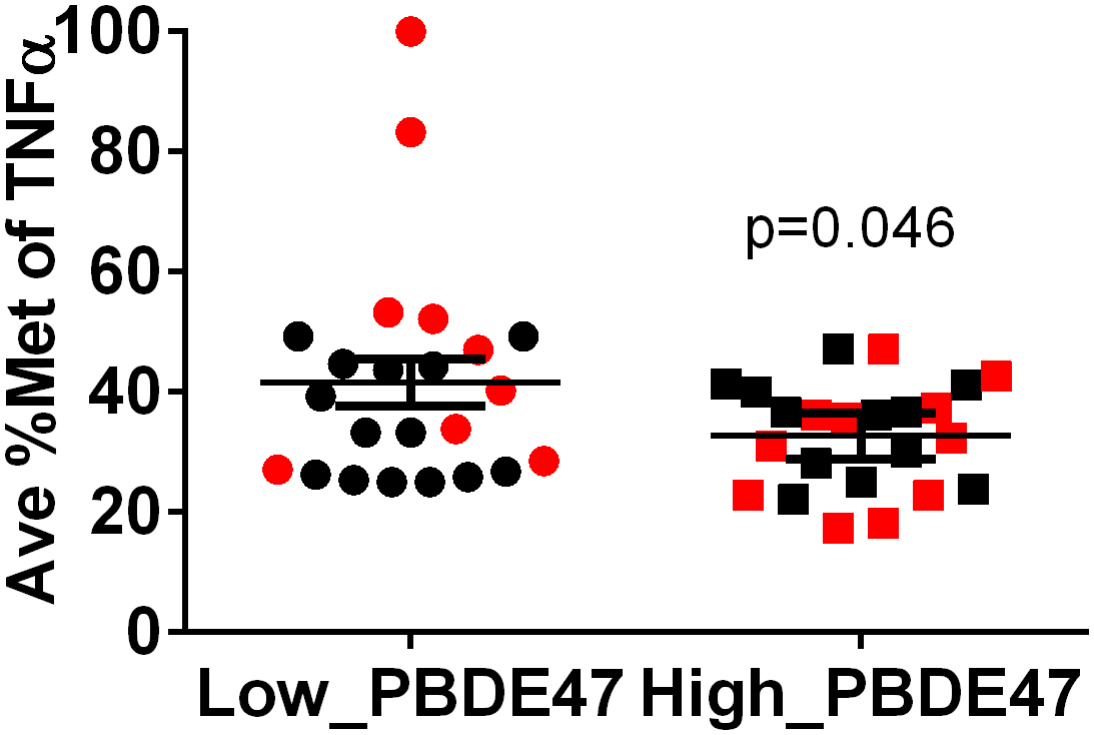
Decreased *TNFα* promoter methylation associates with high maternal PBDE47 exposure. Average % methylation of the *TNFɑ* promoter (y-axis) was calculated by taking an average of the methylation level of a total 12 CpG sites within the *TNFɑ* promoter region in 46 cord blood DNA samples. Results were compared between low- and high-level PBDE47 exposed groups. Each circle and square represented the average percent methylation level for each subject in low-level PBDE47 exposed group and high-level PBDE47 exposed group, respectively. Boys (in black) and girls (in red) are marked differently. The error bars represented means (±standard error of mean, SEM).

**Fig 4:**
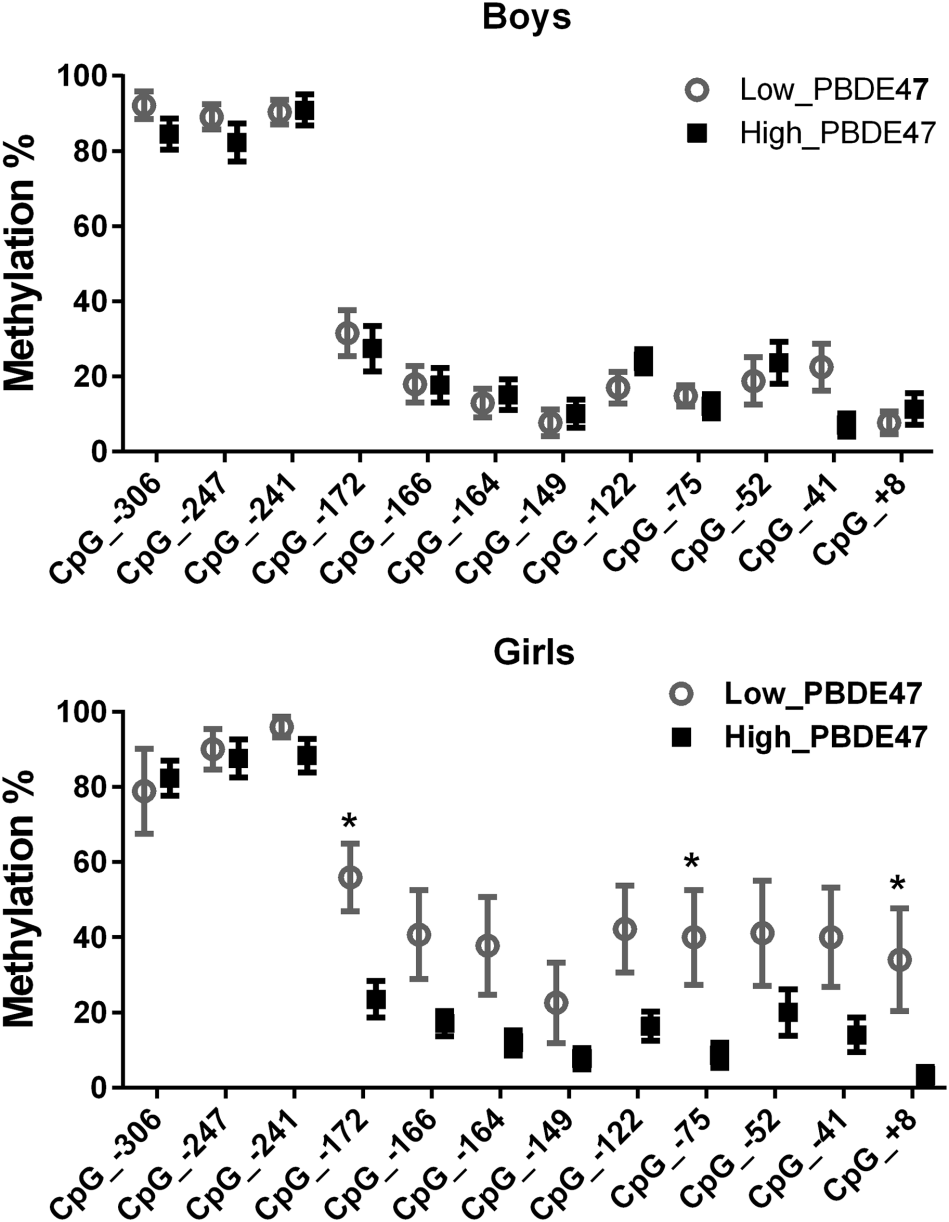
Decreased CpG site-specific methylation of TNFα in cord blood associates with high maternal PBDE47 exposure in girls (lower panel) not boys (upper panel). **Percentage of** methylation of each CpG site of the TNFα promoter represented as mean ± SEM in low- (gray open circles) and high-level (black squares) PBDE47 exposed group. The difference in % methylation in these 12 CpG sites between the two groups was compared by two-way ANOVA. * *p*<0.05, meant % methylation at a CpG site was significantly different between low- and high- level PBDE47 exposed groups (based on t-test).

We analyzed the average % methylation of *TNFɑ* promoter as well as % methylation of each of the 12 CpG sites in the *TNFα* promoter region, separately. % methylation at each CpG site was calculated using the mean value obtained from 4–6 clones for each subject. Average % methylation of the *TNFɑ* promoter was calculated by taking the average of the methylation level of all the analyzed 12 CpG sites within the *TNFɑ* promoter region. A scatter plot with smooth fitted line was generated in R (Version 3.01) to explore the relationship between the average % methylation of *TNFɑ* promoter and log10-transformed maternal blood PBDE47 level (Fig 2). We plotted the distribution of average % methylation of the *TNFɑ* promoter in the low- and high- level PBDE47 exposed groups, separately (Fig 3). We then compared the difference between these two groups using an unpaired t-test (two-tailed p-value) with Welch’s correction. In addition, we generated the plots to describe the distribution of % methylation at each of 12 CpG sites in low- and high-level PBDE47 exposed groups (boys and girls, separately), and then compared the difference between the two groups by two-way ANOVA (Fig 4). We presented *p*-value without adjustment for multiple comparisons due to the fact that 1) the sample size was small; 2) these CpG sites were not independent of each other, in which, Bonferroni adjustment appeared to be very stringent. The difference of cord blood TNFα protein (in log-transformation) between low- and high-level PBDE47 exposed groups was compared using an unpaired t-test. Spearman correlation coefficient was calculated to determine the relationship between cord blood TNFα protein level and average % methylation of *TNFɑ* promoter. Graphs were generated using Prism 6 (GraphPad Software, CA).

## Results

### PBDE47 shows the most abundance among PBDEs congers in BBC

In the BBC cohort, a total of 247 maternal blood samples had available data for blood PBDEs level (of 10 PBDE congeners), which showed a wide range of variability (Table 1). PBDE47 is the most prevalent PBDE (accounting for 58% of the total PBDEs level) in this cohort (Table 1) and thus we focused on the epigenetic effect of PBDE47 in our study. A total of 46 mother-infant pairs (23 in low-level maternal PBDE47 exposed group and 23 in high-level maternal PBDE47 exposed group) were selected for this study (Table 2). There was no significant difference in baby’s gender, smoking status, parity, preterm status, maternal education, maternal age and pre-pregnancy BMI between the selected 46 samples and the rest of the 201 samples (Table 3). Among the 46 selected samples, mothers with high PBDE47 exposure were significantly younger than those with low PBDE47 exposure (p<0.01, Table 3).

**Table 3:** Population Characteristics.

	Low PBDE47 group	High PBDE47 group	p-value*	Enrolled in this study	Non-enrolled in this study	p-value*
N	23	23		46	201	
Boys, N(%)	14(60.9)	11(47.8)	0.554	25(54.3)	99(49.3)	0.646
Maternal smoking during pregnancy, N(%)	2(8.7)	2(8.7)	1	4(8.7)	28(13.9)	0.477
Nulliparty, N(%)	6(26.1)	9(39.1)	0.529	15(32.6)	82(40.8)	0.391
Cesarean section, N(%)	8(36.4)	4(17.4)	0.271	12(26.7)	61(31.8)	0.625
Preterm birth, N(%)	8(34.8)	9(39.1)	1	17(37.0)	106(52.7)	0.077
Maternal education			0.406			0.473
< high school, N (%)	8(34.8)	9(39.1)		17(36.0)	54(27.8)	
High School, N (%)	6(26.1)	9(39.1)		15(32.6)	74(38.1)	
College and above, N (%)	9(39.1)	5(21.7)		14(30.4)	66(34.0)	
Maternal age, year (mean±SD)	31.0±6.6	24.3±5.7	<0.01	27.7±7.0	28.0±6.9	0.78
Maternal BMI, kg/m^2^ (mean±SD)	25.8±5.5	24.9±4.0	0.5	25.3±4.8	25.9±5.5	0.531

SD, standard deviation.

*p values were calculated using Chi-square test and t-test was performed to test group difference for categorical and continuous variables, respectively.

### Decreased cord blood TNFα promoter methylation associated with high maternal PBDE47 exposure

We first utilized an *in vitro* system to test the epigenetic effect of PBDE47 on these cytokines by exposing peripheral blood mononuclear cells (PBMCs) to PBDE47 (0.1–100nM). *TNFα* methylation, not *IL1β*, showed a linear relationship to the doses of PBDEs (data in S1 Fig). DNA methylation patterns of a total of 12 CpG sites located at the promoter region of *TNFα* (Fig 1) were analyzed in cord blood leukocytes. Previous studies have shown the significance of CpG site-specific DNA methylation in *TNFα* transcription although the *TNFα* gene does not have traditional CpG islands (GC%>60%) at its promoter region [39]. We first examined the linear relationship between average % methylation of the *TNFα* promoter in cord blood and maternal blood PBDE47 level (log10-transformed). As shown in Fig 2, their relationship was not linear. Instead, *TNFɑ* methylation remained unchanged when maternal blood PBDE47 level (log10-transformed) was less than 0.7 (or PBDE47 level <5 ng/g-lipid) but *TNFɑ* methylation decreased with increasing maternal PBDE47 exposure when maternal blood PBDE47 level (log10-transformed) is greater than 0.7 (or PBDE level > 5ng/g-lipid).

Compared to that in the low-level PBDE47 exposed group, average % methylation of the *TNFα* promoter was lower in the high-level PBDE47 exposed group (p = 0.046) (Fig 3). When stratified by the gender of offspring, a similar association was found in girls (p = 0.004), but not in boys (p>0.99) (Fig 3). To determine if one or more CpG sites at the *TNFα* promoter were more sensitive to PBDE47 exposure, we compared % methylation at each individual CpG site between low- and high-level PBDE47 exposed groups. In boys, there was no significant difference in % methylation of any CpG sites between low- and high-level PBDE47 exposed groups. In girls, three CpG sites, CpG_-172 (p = 0.04), CpG_-75 (p = 0.05), and CpG_+8 (p = 0.05) showed significantly decreased % methylation in the high-level PBDE47 exposed group as compared to that of low-level PBDE47 exposed group (Fig 4).

### Inverse relationship between cord blood *TNFα* promoter methylation and cord blood TNFα protein level in girls

To examine if the decreased cord blood *TNFα* promoter methylation contributed to increased gene expression, we measured TNFα protein level in 23 (out of 46 selected samples) cord blood serum as RNA sample was not available. The 23 selected samples were not chosen by either maternal PBDE47 level or cord blood methylation status. The analysis of TNFα protein level in serum was primarily designed to study preterm birth and related traits in other BBC-related studies. Therefore, we would expect more cases of preterm birth among the 23 selected samples we measured cord blood TNFα protein. When compared with those unselected samples (without TNFα protein analysis), these 23 selected samples were more apt to preterm birth (65.2% vs 8.7%, p = 0.0003). Nonetheless, they were comparable with unselected samples on other variables, such as, gender, nullipartiy, cesarean section, maternal education, maternal age and maternal BMI. The distribution of cord blood TNFα protein level in each PBDE47 exposed group was shown in Table 4. The median of TNFα protein level in the low-level PBDE47 exposed group (n = 13) (28.2 pg/mL) was higher than that of high-level PBDE47 exposed group (n = 10) (19.3pg/mL). A similar trend was found in boys (n = 15). In girls (n = 8), cord blood TNFα protein level tended to be higher in the high-level PBDE47 exposed group than in the low-level PBDE47 exposed group. However, none of the differences were statistically significant.

**Table 4:** Distribution of cord blood TNFα protein level, stratified by maternal blood PBDE47 level.

	TNFα (pg/mL)*, Median (P25-P75)		
	Low PBDE47 group	High PBDE group	p-value^b^
Total Sample (n^a^ = 13 / 10)	28.2 (13.5–36.1)	19.3 (13.4–31.8)	0.75
Boys (n^a^ = 10 / 5)	31.5 (16.5–40.5)	13.9 (13.4–26.0)	0.18
Girls(n^a^ = 3 / 5)	9.0 (6.6–22.6)	23.7(14.8–37.2)	0.39

*The 23 selected samples were not chosen by either maternal PBDE47 level or cord blood methylation status. The analysis of TNFa protein level in serum was primarily designed to study the preterm birth and related traits in other BBC-related studies.

^a^ Sample size in low-level PBDE47 exposed group /in high-level exposed PBDE47 group

^b^ *p* values were estimated based on Welch two-sample t-test for log-transformed cord blood TNFα levels between low- and high-level PBDE47 exposed groups.

As shown in Table 5, average % methylation of the *TNFα* promoter was not significantly correlated to cord blood TNFα protein level in 23 samples, as indicated by the Spearman’s rank coefficient (ρ, rho). When we focused this analysis to each specific CpG site at *TNFα* promoter, we did not identify any significant association in the total 23 samples and in boys (n = 15). In girls, we found that % methylation at CpG_+8 negatively correlated with cord blood TNFα level (ρ = -0.733, *p*-value 0.039). It suggested increases in cord blood TNFα protein level associates with hypomethylation at a specific CpG site (CpG_+8) at the *TNFα* promoter.

**Table 5:** Spearman correlation between cord blood TNFα promoter methylation and cord blood TNFα protein level.

		Total = 23		Boys, N = 15		Girls, N = 8	
CpG loci_from TSS	Genome Coordinate at NC_000006.12	rho*	p-value*	rho*	p-value*	rho*	p-value*
CpG_-306	31575258	0.333	0.12	0.311	0.259	0.279	0.503
CpG_-247	31575317	-0.242	0.266	-0.045	0.874	-0.504	0.203
CpG_-241	31575323	-0.132	0.575	-0.207	0.46	-0.055	0.898
CpG_-172	31575392	0.032	0.886	0.199	0.476	-0.419	0.301
CpG_-166	31575398	0.015	0.945	0.096	0.732	-0.074	0.862
CpG_-164	31575400	0.048	0.828	0.215	0.442	-0.388	0.342
CpG_-149	31575415	0.203	0.352	0.141	0.616	0.077	0.857
CpG_-122	31575442	0.039	0.858	-0.017	0.953	-0.026	0.952
CpG_-75	31575489	-0.19	0.386	-0.14	0.62	-0.25	0.55
CpG_-52	31575512	-0.152	0.49	-0.21	0.452	-0.024	0.954
CpG_-41	31575523	-0.06	0.786	-0.055	0.846	-0.146	0.729
CpG_+8	31575572	-0.2	0.36	0.112	0.691	-0.733	*0.039
Total CpGs	31575258–572	0.005	0.98	0.075	0.79	-0.238	0.57

*The Spearman’s rank correlation coefficient (ρ, rho) was calculated to measure the strength of the relationship between the percent of methylation of *TNFα* promoter and TNFα protein level in cord blood samples. Negative values indicated inverse relationship between cord blood *TNFα* promoter methylation and cord blood TNFα protein level. The larger the negative value meant greater negative correlation. Statistical significance at individual CpG site was determined with *p*<0.05.

## Discussion

Our results suggested that maternal PBDE47 exposure altered CpG site specific DNA methylation at the *TNFα* promoter, which may contribute to the aberrant TNFα gene expression in the offspring. There are intensive studies that have reported the toxic effect of PBDEs on neural and thyroid systems [7, 12–13]. Strikingly, PBDEs can also influence the functions of the immune system [14, 28–29]. Our previous studies reported a number of CpG sites (located within the genes with immunological functions) showing significant longitudinal changes in methylation levels [34]. Recently, we have demonstrated that pre-pregnancy maternal BMI associated with the epigenetic alterations in offspring’s genes related to the inflammatory and lipid metabolism response [40]. Here, we provide the evidence showing the relationship between maternal PBDE47 exposure and gene-specific methylation of a proinflammatory gene at birth. TNFα is a proinflammatory cytokine produced mainly by macrophages but also found in T cells, B cells and fibroblasts. This cytokine has been implicated in a variety of diseases including immune-related disease [41–43] and cancer [44]. The proximal region of the *TNFα* promoter (~200 bp upstream of the TSS) regulates the transcription of *TNFα* in multiple cell types that respond to various stimuli including the T cell and B cell activation, infection and cytokines [45]. Sullivan *et al* demonstrated that TNFα production correlated to the CpG site-specific methylation of the *TNFα* promoter in monocytes and THP-1 cells in the presence of lipopolysaccharide (LPS) [39]. By using a subset of the BBC, we showed that *TNFα* hypomethylation in cord blood correlated to high maternal PBDE47 exposure. Remarkably, we found that girls are more susceptible to *TNFα* promoter hypomethylation in response to maternal PBDE47 exposure. Further, we showed, in girls, *%* methylation at the specific CpG site (CpG_-172, CpG_-75 and CpG_+8) as previously described by *Sullivan et al* [39], significantly associated with maternal PBDE47 exposure. In addition, methylation status of CpG_+8 (girls) inversely correlated to TNFα protein level in cord blood. It suggests that maternal PBDE47 exposure might alter TNFα production in the offspring via CpG site-specific DNA methylation. We did not note a statistically significant correlation between cord blood TNFα protein level and maternal PBDE47 exposure in girls, although we found the median of TNFα protein level in the high-level PBDE47 exposed group was higher than that of low-level PBDE47 exposed group. We acknowledged that this TNFα protein analysis is limited by a relatively small sample size and so these findings warrant further validation in other samples enrolled in BBC.

Studies showed that transcriptional control at the *TNFα* promoter associated with its production and with the disease risk. In Jurkat T cells or human monocytic cells, promoter methylation [39, 46], histone acetylation and methylation [47–49] together with other transcription factors [50] could regulate transcription of *TNFα* gene. Long noncoding RNAs and their binding proteins were shown to regulate gene expression of TNFα [51]. Histone modifications at the TNF promoter also associated with the disease states of diabetes [52] and systemic lupus erythematosus [53]. Women with higher adiposity showed decreased methylation of the *TNFα* promoter and higher plasma TNFα protein level in their peripheral white blood cells [54]. Further, epigenetic regulation of the monocyte and T cell lineages by TNFα production showed to play a key role in immunological diseases [55]. These results suggested that epigenetic regulation of TNFα may contribute to the modulation of inflammatory responses.

No studies have examined the association between the maternal PBDE exposure and TNFα promoter methylation in the developing fetus. Newborns with growth restriction showed increased TNFα expression in the placenta [56]. It suggests that TNFα cytokine expression may influence developmental outcomes of the offspring. Our study, although limited by a small sample size and no adjustment for multiple testing, may represent the first step to demonstrate the potential influence of prenatal PBDE exposure on the fetal epigenome. We acknowledged that cord blood leukocytes utilized in the current study consist of many functionally and developmentally distinct cell populations in varying proportions. Because DNA methylation is cell-type specific, it is possible that cellular heterogeneity may confound our methylation measurements. It suggests that differential cell counting and/or sorting of cells from whole blood is required to adjust the findings from the methylation patterns by regression model (using the estimated cell type composition as a covariate in the regression model [57]) or access the methylation patterns in different types of blood cells [58, 59]. Unfortunately, it is not feasible to obtain the cell distribution of the immune cells in the archived samples we utilized in this study. Given the fact that cord blood leukocytes may contain stem cells that can populate the brain in later life [60] and also provide a rich source of immune cells which are important producers of cytokines [61], it suggests that cord blood leukocytes (if only archived cord blood samples are available) may provide a reasonable surrogate for our target organ/tissues (brain/immune cells). Our previous study demonstrated the feasibility of using cord blood DNA methylation as a surrogate biomarker to associate the risk of childhood asthma [21, 23]. All-in all, in the future, we will take this step further by performing replicated studies in a larger sample pool and by following up the developmental outcomes of the offspring from infancy, childhood, to adolescence. We will assess the disease phenotypes like food allergy, asthma as well as metabolic and neurological disorders. If we further confirm our findings on a relatively large sample size at the BBC, we may be able to evaluate the use of cord blood TNFα methylation patterns as biomarkers for the susceptibility of immunological and neurological diseases. Or we can measure cord blood TNFα methylation to assess the adverse health effects of maternal PBDEs exposure.

In the present study, we adjust the BBC samples by mother’s age and birth implications and outcomes (low birth weight). Nevertheless, profound factors for TNFα methylation may include social-economics status, psychological stress, ethnicity, and exposures to other endocrine disrupting chemicals and PBDEs congers. PBDE47 showed sex-, age-, species- and dose-dependent effects on its metabolism and elimination in experimental animal studies [62–65]. Therefore, we must carefully choose proper data modeling and multiple testing when we employ the future epigenetic analyses. We will need a set of follow-up studies, *in vitro* or *in vivo*, to show the epigenetic effect of the PBDEs toxicity in humans. Taken together, our findings provide evidence that maternal exposure to PBDEs may induce epigenetic reprogramming of the offspring’s immune response by aberrant DNA methylation of a proinflammatory gene.

## Supporting Information

S1 Fig Exposure to PBDE47 decreased TNFɑ but not IL1ß methylation status of peripheral blood mononuclear cells. Peripheral blood mononuclear cells (PBMCs, HemaCare, CA) were exposed to a range of PBDE47 (AccuStandard, NH) exposures for 3 days. DNA isolated from cells was undergone bisulfite treatment before PCR. Promoter methylation status for TNFɑ and IL1ß was assayed by bisulfite genomic sequencing. Results were collected from three independent sets of experiment and analyzed by 2-tailed ttest. Error bar represented standard error of mean. *p*<0.05 presented result was statistically significant in PBDE47-exposed group when compared with that of untreated control (PBDE47_0nM).
(TIFF)
